# Biodegradation of 4-chlorophenol in an airlift inner loop bioreactor with mixed consortium: effect of HRT, loading rate and biogenic substrate

**DOI:** 10.1007/s13205-016-0435-5

**Published:** 2016-06-02

**Authors:** Bhishma P. Patel, Arvind Kumar

**Affiliations:** Environmental Pollution Abatement Lab, Chemical Engineering Department, National Institute of Technology, Rourkela, Odisha 769008 India

**Keywords:** 4-chlorophenol, Airlift reactor, Biodegradation, Wastewater, Biogenic substrate

## Abstract

In the present work, removal of 4-chlorophenol (4-CP) by the mixed microbial consortium was evaluated in an airlift inner loop bioreactor. During the study, the effect of various reactor parameters such as hydraulic retention time (HRT), biogenetic substrate concentration, loading rate, and initial substrate concentration on the removal efficiency of 4-CP was investigated. Bioreactor showed a maximum removal rate of 16.59 mg/L/h at the optimum conditions of 24 h HRT, 400 mg/L initial 4-CP, and 0.2 g/L peptone. The optimum HRT found was 24 h after that the washout occured, and the degradation efficiency almost dropped to 50 % at 18 h HRT. Effect of peptone showed that lower concentration of peptone improves 4-CP removal efficiency of the bioreactor. Also, the mixed consortium had utilized 4-CP as a carbon source, as evidenced by the increasing biomass concentration with 4-CP at constant peptone concentration. The presence of 5-chloro 2-hydroxymuconic semialdehyde in the reactor infers that the mixed consortium has followed the meta-cleavage pathway for 4-CP degradation.

## Introduction

With heavy industrialization and urbanization, activities polluting the environment and concomitantly sabotaging the inherent ecosystems have enormously increased. Chlorophenols have been classified as priority pollutants by the US Environmental Protection Agency (ATSDR 2015). They are primarily found in the industrial effluent from pulp and paper, leather tanning, biocide, dyes, herbicide and chlorination of drinking and waste water (Olaniran and Igbinosa 2011; Field and Sierra-Alvarez 2008; Wang et al. 2014). Chlorophenols are persistent and bio-accumulative in nature due to its physicochemical properties that cause detrimental effects on the environment. Chlorophenol compounds have been demonstrated to have mutagenicity, carcinogenicity, immunogenicity, and may cause fatalities (IARC 1986; Daniel et al. 2001). Conventional methods such as adsorption, chemical oxidation, photo-degradation and solvent extraction have limitations due to their high cost of implementation, production of toxic by-products and low energy efficiency (Herrera et al. 2008; Durruty et al. 2011). These treatments transform the toxic compounds from one phase to another. On contrary, biological treatment is a potential substitute with promising removal efficiency and complete mineralization of the chlorophenols. It is eco-friendly and energy efficient treatment with assuring future (Field and Sierra-Alvarez 2008; Olaniran and Igbinosa 2011; Basak et al. 2013, 2014; Chakraborty et al. 2013).

There are several reports on various microorganisms that have been identified for utilizing chlorophenols as a carbon and energy source such as *Pseudomonas aeruginosa, Pseudomonas putida, Comamonas testosteroni, Ralstonia eutropha, Bacillus subtilis, Arthrobacter chlorophenolicus, Burkholderia* sp., and *Kocuria rhizophila*. (El-Sayed et al. 2009; Arora and Bae 2014; Field and Sierra-Alvarez 2008; Hollender et al. 1997; Monsalvo et al. 2012; Patel and Kumar 2015, 2016). Biodegradation of chlorophenols in different bioreactor system under aerobic and anaerobic conditions has been reported. Attention has been given to study and modify the different bioreactors such as packed bedreactor, fluidized bed reactor, upflow anaerobic sludge blanket, combined anaerobic and aerobic treatment and membrane reactor to enhance the performance of wastewater treatment containing lower to higher chlorophenols (Quan et al. 2003; Galíndez-Mayer et al. 2008; Gomez-De Jesus et al. 2009; Visvanathan et al. 2005; Atuanya and Chakrabarti 2004). Aerobic biodegradation is usually preferred for lower chlorophenols such as mono and di-chlorophenol and sometimes tri-chlorophenols. While, for the treatment of higher chlorophenols such as tri, tetra, and pentachlorophenol, an anaerobic biodegradation is more effective (Poggi-Varaldo et al. 2012).

Wastewater treatment plant commonly contains the mixture of different recalcitrant synthetic organic compounds (SOC) along with biogenic substrates. The common treatment plants are not able to treat recalcitrant SOC effectively and generally passes through without complete treatment (Hu et al. 2005). The treatment of recalcitrant compounds can be improved by process optimization. These require the understanding of biomass acclimation, interaction between biogenic substrate and recalcitrant compounds, and reactor operating conditions for SOC treatment. Different factors such as hydraulic retention time, loading rate and the presence of other secondary carbon and nitrogen source have to be considered and optimized for better performance and efficiency of the bioreactors.

Airlift reactors (ALR) are simple in design with no moving parts or agitator, thus economical. In ALR, pressurized gas is used for internal mixing and aeration. It has low shear stress on microbial cells which will be an advantage for shear sensitive cells. In packed bed reactor where mass and oxygen transfer limitation and clogging of packing bed occur, these problems can be avoided in ALR (Zilouei et al. 2006; Quan et al. 2003). There are several reports on biodegradation of chlorophenols and other toxic compounds using ALR. Quan et al. (2003, 2004) reported the removal of phenol and 2,4-dichlorophenol in the airlift honeycomb-like ceramic reactor (Quan et al. 2003, 2004). They have used ceramic honeycomb-like structure placed inside the riser for immobilization of pure culture *Achromobacter* sp. A packed bed bioreactor equipped with the net draft tube riser (PB-ALR) for liquid circulation and oxygenation was constructed for the removal of 2,4,6-trichlorophenol (Gomez-De Jesus et al. 2009). In the present study, simple airlift bioreactor without any immobilization of microorganism was used for removal of 4-chlorophenol. The absence of agitator for mixing and any carrier for immobilization of microorganism makes the bioreactor simple with ease in operation and economical.

The present study focussed on the continuous biodegradation of 4-chlorophenol in an airlift inner loop bioreactor using the mixed microbial consortium. During the continuous operation, the effect of different parameters such as hydraulic retention time, loading rate, biogenic substrate concentration and initial substrate concentration on removal efficiency of ALR was studied. The different metabolites present in the effluent were detected to analyze the biodegradation pathway followed by the mixed consortium.

## Materials and methods

### Chemicals and reagents

Loba chemie, India supplied analytical grade 4-CP (purity 98 %). The stock solution of 4-CP was prepared in 0.02 M NaOH and pH was adjusted to 7.4 ± 0.2 by 1 M orthophosphoric acid. All other inorganic chemicals used in the experiments were of analytical grade and obtained from Merck, India. HPLC grade reagents were obtained from Hi-media, India for HPLC analysis.

### Microorganisms

The mixed microbial consortium used in the study was previously isolated from the sludge, and soil samples collected from the dye industries effluent treatment plant, Gujarat, India. For enrichment and acclimatization, 10 gm of sludge and soil was added to 100 mL of mineral salt medium (MSM) (composition mentioned elsewhere in the paper) containing peptone (1–0.1 g/L) and 2,4-DCP (20–250 mg/L) and incubated at 30 °C and 120 rpm in rotary shaker for a period of 6 months. The enriched culture was transferred to fresh MSM at every 15 days with increasing concentration of 2,4-DCP. The mixed consortium resulted after acclimatization period is used in the present study for biodegradation of 4-CP.

### Bioreactor medium

The bioreactor was fed with the MSM containing 2,4-DCP and peptone as a carbon and energy source. The composition of the MSM (modified DSMZ-465) was (g/L): Na_2_HPO_4_·2H_2_O 3.5, KH_2_PO_4_ 1, (NH_4_)_2_SO_4_ 0.5, MgCl_2_·6H_2_O 0.1, NaNO_3_ 0.05 and 1 mL of trace element solution having composition of (g/L): EDTA 0.5, FeSO_4_·7H_2_O 0.2, CuCl_2_·2H_2_O 0.001, ZnSO_4_·7H_2_O 0.01, MnCl_2_·4H_2_O 0.003, CoCl_2_·6H_2_O 0.02, H_3_BO_3_ 0.03, Na_2_MoO_4_·2H_2_O 0.003. The trace elements and 4-CP were added to the medium after autoclaving by filter sterilizing using 0.22 µm filters. The pH of the medium was adjusted to 7.35 ± 0.1 using 1 M H_3_PO_4_ and 1 M NaOH. For the bioreactor study, the medium was prepared using the filtered tap water.

### Bioreactor

**Fig. 1:**
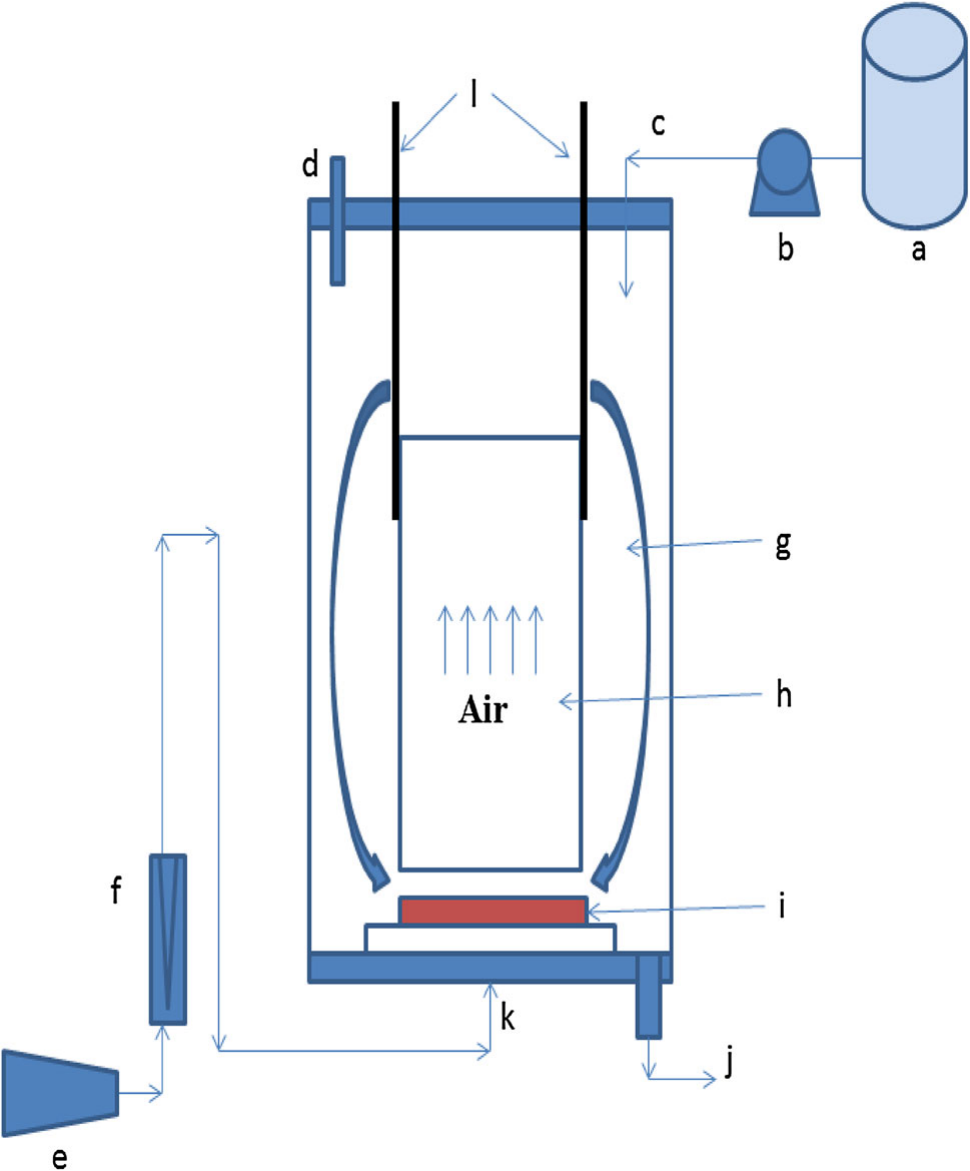
A schematic view of an airlift inner loop bioreactor. **a** Feed tank, **b** peristaltic pump, **c** influent, **d** air outlet and pH probe, **e** air compressor, **f** air rotameter, **g** downcomer, **h** riser, **i** air diffuser, **j** drainage or effluent, **k** air input, **l** metal rod supporting inner draft tube

An airlift inner loop reactor made of Perplex glass was used throughout the experiment (Fig. 1). The outer core has a dimension of 18 × 60 cm with a concentrically located inner draft tube of 9.5 × 35 cm dimension. The working volume of the reactor is 12 L. The inner draft tube was supported by three metallic clamps. The ceramic disc (90–150 µm pore size) with 9 cm diameter located below the inner tube was used as air sparger. The medium was fed to the bioreactor using gravitational flow and the flow rate was controlled by the peristaltic pump. Air flow rate used during the study was 4 LPM. The bioreactor was stored in an isolated room with temperature controlling facility (32 ± 3 °C).

The present investigation focussed on continuous removal of 4-CP, divided into three parts. First, the effect of peptone concentration on removal efficiency of 4-CP was studied. Second, the effect of substrate concentration was studied by gradually increasing it and keeping the other parameters constant. Finally, the effect of HRT on 4-CP removal efficiency was evaluated by gradually decreasing HRT and keeping 4-CP and peptone concentration constant.

### Analytical methods

Biomass concentration was determined by measuring optical density at 600 nm by UV–Visible spectrophotometer (Shimadzu UV-1800, Japan) (Ziagova and Liakopoulou-Kyriakides 2007). The residual concentration of 4-CP was determined by HPLC system (Jasco, US) coupled with MD-2015 photodiode array detector and 2080 plus isocratic pump. The one mL sample was centrifuged at 10,000 rpm for 10 min and supernatant is filtered through 0.22 µm filter before analysis. The sample volume taken was 20µL. The column used was Agilent TC-C18 (25 mm × 4.6 mm); the sample was eluted at a flow rate of 0.75 mL/min with mobile phase consisting of methanol:water:acetic acid (80:19:1 %); detection wavelength is 280 nm. The 4-CP degradation (%) was calculated by analyzing the area under the curve (Sahinkaya and Dilek 2007; Karn et al. 2010).

The presence of 5-chloro-2-hydroxymuconicsemialdehyde (5-CHMS) was determined at 380 nm UV–Visible spectrophotometer (Shimadzu UV-1800, Japan) (Farrell and Quilty 1999).

Catechol and chlorocatechol were analyzed using the method of Arnow (1937). The samples were centrifuged at 10,000 rpm for 10 min. The supernatant (0.5 mL) was treated with 0.5 N HCl (0.5 mL). After mixing, 0.5 mL of nitrite molybdate reagent was added to it resulting in a yellow color. Nitrite molybdate reagent was prepared by dissolving 10 gm of sodium nitrite and 10 gm of sodium molybdate in 100 mL of distilled water. After mixing, 0.5 mL of 1 N NaOH was added to the mixture resulting in a red color. Finally, the absorbance was measured at 510 nm.

## Results and discussion

The bioreactor was operated for 60 days in a continuous mode during which the effect of different parameters such as HRT, peptone concentration and loading rate on the removal of 4-CP studied. The bioreactor showed an excellent removal efficiency of 4-CP during continuous operation.

Before the commencement of the continuous operation, the reactor was operated without inoculation to check the abiotic loss of the 4-CP due to evaporation. The bioreactor was filled with MSM containing 40 mg/L of 4-CP for 1 day, and the air flow was set to 4 LPM. At these conditions, 2–3 % loss due to evaporation by air flow was observed which is negligible as compared to biodegradation.

The bioreactor was filled with 12 L MSM containing 20 mg/L of 4CP and inoculated with 10 % (v/v) mixed consortium. The bioreactor was operated in the fed-batch mode before the continuous study to acclimatize the biomass. The 4-CP was added to bioreactor periodically to maintain the 20 mg/L concentration. This acclimatization process was performed for one week and afterwards the continuous operation was started.

### Effect of peptone

**Fig. 2:**
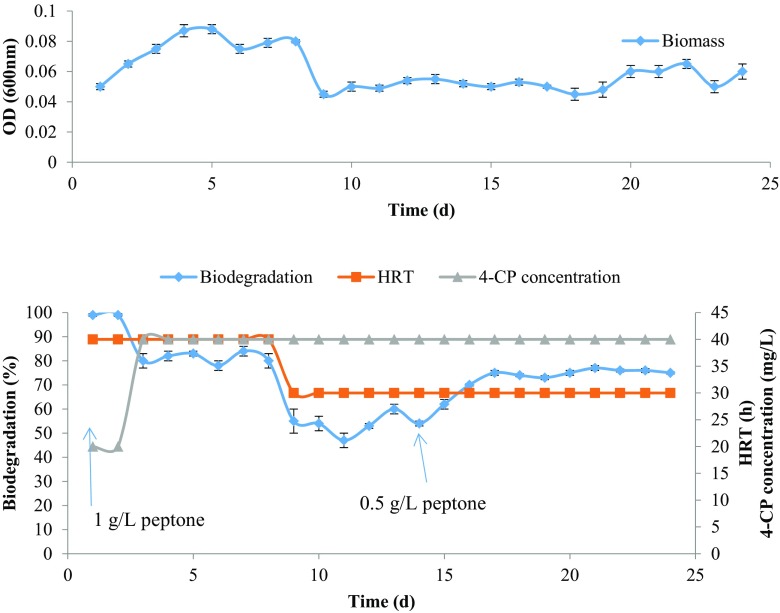
The 4-CP removal by ALR and the effect of peptone concentration. The above figure shows the change in biomass concentration in the effluent during the continuous operation

**Fig. 3:**
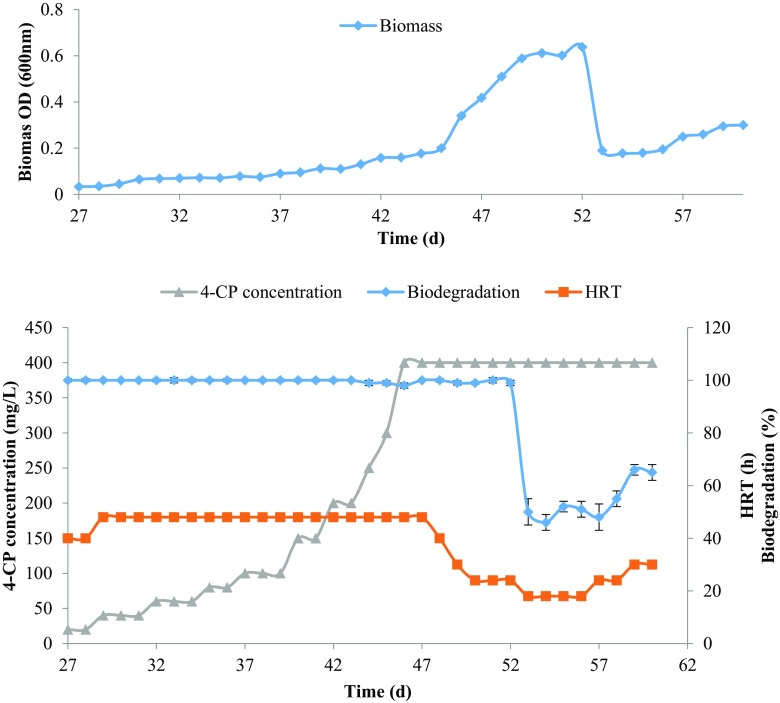
The 4-CP removal efficiency by ALR in the presence of 0.2 g/L peptone and effect of initial substrate concentration and HRT. The above figure shows the change in biomass concentration in the effluent during the continuous operation

The effect of peptone on the removal of 4-CP in ALR was studied throughout the operation at different operating conditions. The bioreactor was started with 1 g/L peptone with HRT of 40 h. The effect of peptone in the removal of 4-CP by ALR is presented in Figs. 2 and 3. The bioreactor showed 99 % removal of 20 mg/L of initial 4-CP present in the influent. However, upon increasing the initial 4-CP concentration to 40 mg/L, the removal efficiency was decreased to around 80 % at 40 h HRT. At this condition, when HRT was reduced to 30 h, the removal efficiency was decreased to around 50 % and got stabled at 60 % with 1 g/L peptone. The reduction in removal efficiency can be explained by two possible reasons. First, at lower HRT, dilution rate was increased which leads to washout and reduction of biomass concentration in the medium. Second, at lower HRT, peptone loading rate was increased leading to decreased level of competent biomass in the medium.

On 15th day of the operation, the peptone concentration was decreased to 0.5 g/L in the influent by keeping initial 4-CP concentration at 40 mg/L and HRT at 30 h (Fig. 2). The bioreactor showed an increase in removal efficiency from 60 to 76 % in the presence of low peptone concentration at steady-state condition. On 27th day of the operation, the peptone concentration was further decreased to 0.2 g/L in the influent. The HRT was set to 48 h and 4-CP concentration in the influent was increased gradually at this condition. The bioreactor had shown complete removal of 40 mg/L 4-CP. The bioreactor performance had greatly increased in the presence of 0.2 g/L peptone. Even the bioreactor was able to remove up to 400 mg/L of 4-CP in the influent with greater than 98 % removal efficiency. It was observed that the biomass concentration in the medium significantly increased at lower peptone concentration. The result indicates that the mixed consortium can utilize the 4-CP as a carbon source with complete mineralization.

The presence of higher concentration of peptone interferes with the degradation of 4-CP by the mixed consortium. However, at low peptone concentration, the removal efficiency increased but some peptone was found to be necessary to maintain the removal efficiency. Same results were obtained in batch studies which also showed that the presence of 0.2 g/L of peptone improves the degradation efficiency compared to the absence of peptone. This can be concluded as the peptone served as a nitrogen source to the mixed consortium as it was used during the acclimatization period. Also, the peptone did not contribute to biomass growth as evidenced by the result that the biomass increases with the decrease in peptone concentration and increase in 4-CP concentration. Thus, it could be concluded that the mixed consortium utilized the 4-CP as a carbon source. Table 1 summarizes the effect of peptone on the removal rate of 4-CP by the mixed consortium.

**Table 1:** Effect of peptone concentration on 4-CP removal by ALR

Time (day)	Influent concentration (mg/L)	HRT ± 1 (h)	Loading rate (mg/L/day)	Peptone (mg/L)	Biodegradation (%)	Volumetric removal rate (mg/L/h)
1–2	20	40	12	1	99	0.495
3–8	40	40	24	1	82	0.82
9–14	40	30	32	1	56.5	0.753
15–24	40	30	32	0.5	75.8	1.01
29–31	40	48	20	0.2	100	0.84
42–43	200	48	100	0.2	100	4.17
44	250	48	125	0.2	99	5.2

A specific group of microorganism in the mixed consortium was responsible for the degradation of 4-CP. Sahinkaya and Dilek (2006) studied the effect of peptone on the degradation of 4-CP in sequencing batch reactor using a mixed microbial consortium and reported that competent biomass (specialist) is responsible for 4-CP degradation and so the specific degradation rate of 4-CP increases with decreasing peptone concentration as the fraction of competent biomass increases in the mixed consortium (Sahinkaya and Dilek 2006). There are several reports on the degradation of chlorophenols in the presence of other carbon and nitrogen source such as glucose, dextrose, peptone, and yeast extract. The presence of secondary carbon and nitrogen source witnessed an increase in the degradation of chlorophenols (Sahinkaya and Dilek 2006; Murialdo et al. 2003; Shen et al. 2005; Kim et al. 2002; Wang and Loh 1999). However, there are others reports that publicized the opposing outcomes that sometimes the degradation of higher chlorophenols were inhibited (Durruty et al. 2011; Alexander 1999). Shen et al. (2005) had described the beneficial nature of the presence of a suitable quantity of microbial easily degradable substrate for stimulating the process of dechlorination and degradation of chlorophenols (Shen et al. 2005).

### Effect of substrate concentration

Effect of initial 4-CP concentration on the removal efficiency of bioreactor was studied by keeping the HRT (48 ± 1 h) and peptone concentration (0.2 g/L) constant and gradually increasing 4-CP concentration. The bioreactor showed greater than 99 % removal efficiency when the initial 4-CP concentration was increased from 20 to 400 mg/L. The effect of initial substrate concentration on biodegradation is shown in Fig. 3 (Day 27–47). After 150 mg/L, the 4-CP concentration was increased at a fast rate (150–400 mg/L) to check the bioreactor stability for shock loads. The bioreactor has shown great stability and removal efficiency even during the shock loads. At lower concentration of 4-CP, complete mineralization was observed with no traces of the intermediate compounds. However, at higher concentration (above 250 mg/L), the trace amount of 5-chloro 2-hydroxymuconic semialdehyde (5-CHMS) has been observed. The 5-CHMS (*λ*_max_ = 380 nm) is the meta-cleavage product of the 4-chlorocatechol that gives a characteristic yellow color in the medium. The bioreactor medium was turned to light yellow color when 4-CP concentration was increased to 400 mg/L due to the accumulation of 5-CHMS. Also, the biomass concentration in the medium was observed to increase with increasing 4-CP concentration. These results indicate that the microorganisms were able to utilize the 4-CP as a sole carbon source.

### Effect of HRT

**Fig. 4:**
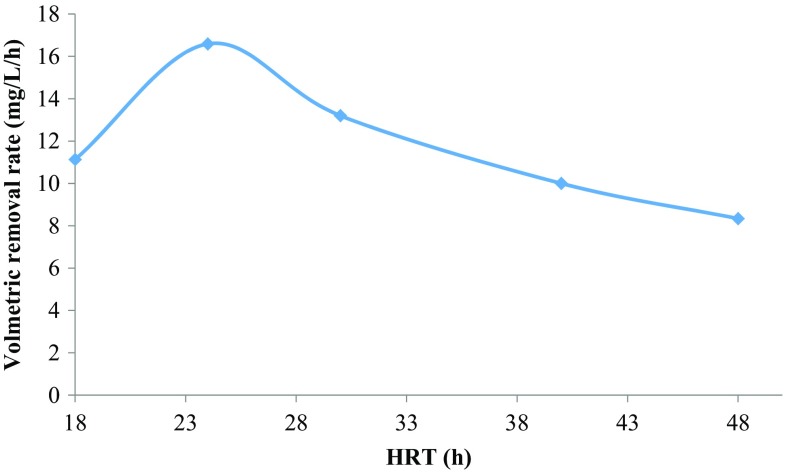
Effect of HRT on the volumetric removal rate of 4-CP (400 mg/L) in the ALR with 0.2 g/L of peptone

Effect of HRT on the removal of 4-CP by ALR has been studied from days 47 to 60. The peptone and initial 4-CP concentration was kept constant at 0.2 and 400 g/L, respectively. The change in the biodegradation of 4-CP at different HRT is shown in Fig. 3. When the HRT was gradually decreased from 48 to 24 h, no abrupt change in the removal efficiency has been observed. The bioreactor showed greater than 99 % removal of 4-CP at 24 h HRT. The presence of 5-CHMS was not observed in the effluent at higher HRT. However, further decrease in HRT to 18 h leads to drastic decrease in biodegradation rate to 50 %. At 18 h HRT, biomass concentration in the bioreactor medium was suddenly decreased as washout occurs and biomass growth rate was decreased. Also, the bioreactor medium has been found to turn into light brown color and 4-CP concentration in the effluent increases suddenly. Effect of different HRT on the volumetric removal rate of 4-CP (400 mg/L) is shown in Fig. 4. The HRT was again increased stepwise to 24 and 30 h to regain the biodegradation capacity of the reactor. The bioreactor takes some time to recover full degradation efficiency. At 24 h HRT, there was a slight increase in biodegradation rate observed. The biodegradation rate was gradually increased to 66 % at steady state when HRT was increased to 30 h. With time and increase in HRT, the bioreactor had showed to regain biodegradation capacity once again. Table 2 summarizes the change in volumetric removal rate with HRT and initial substrate concentration.

**Table 2:** Removal of 4-CP by ALR in the presence of 0.2 g/L peptone

Time (day)	Influent concentration (mg/L)	HRT ± 1 (h)	Loading rate (mg/L/day)	Biodegradation (%)	Volumetric removal rate (mg/L/h)
45	300	48	150	99	6.19
46–47	400	48	200	100	8.34
48	400	40	240	100	10
49	400	30	320	99	13.2
50–52	400	24	400	99.5	16.59
53–56	400	18	534	50	11.13

**Fig. 5:**
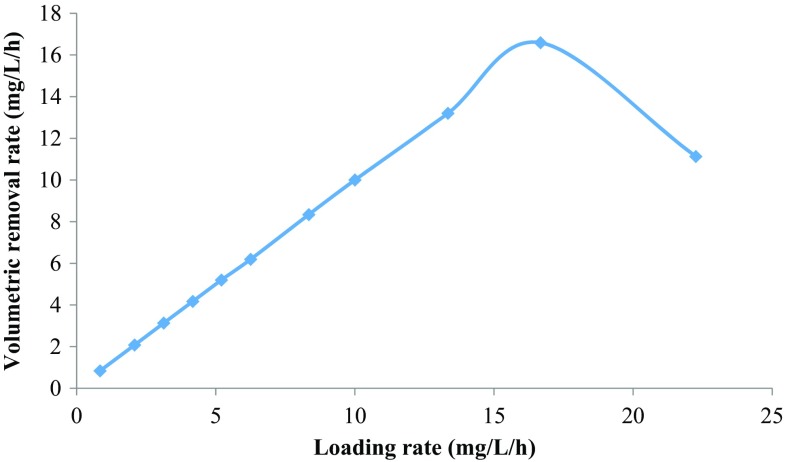
Effect of loading rate on the volumetric removal of 4-CP in the ALR with 0.2 g/L of peptone

The relationship between volumetric loading rate and volumetric removal rate is depicted in Fig. 5. The removal rate was increased exponentially with loading rate up to 16.67 mg/L/h, where 16.59 mg/L/h volumetric removal rate was observed. However, when loading rate was increased to 22.25 mg/L/h, there was a drastic reduction in volumetric removal rate observed as shown in Fig. 5. So, for achieving maximum or greater than 98 % removal rate, the HRT should be kept at 24 h and loading rate at 16.67 mg/L/h. Kargi and konya (2007) studied the effect of HRT on the removal of 4-CP in activated sludge unit. They have reported volumetric removal rate in the range of 360–720 mg/L/day with high COD for different HRT of 5–15 h. After 15 h of HRT, there was no significant increase in the removal rate observed (Kargi and Konya 2007). In another study, it was reported that degradation of 4-CP was decreased with a decrease in HRT in UASB. The UASB had shown 90.1, 88.3, 84.6, and 83 % degradation of 4-CP at 16, 12, 8, and 6 h, respectively (Majumder and Gupta 2008). Table 3 shows the performance of different bioreactors for removal of chlorophenols where most of the studies had shown the removal of 4-CP below 200 mg/L of loading rate. Kargi and Konya (2007) had shown the removal of 4-CP up to 800 mg/L of loading rate with 90 % efficiency (Kargi and Konya 2007). In the present study, the bioreactor had achieved 99.8 % removal for higher loading rate of 400 mg/L/day. The performance of the ALR for removal of 4-CP is prominent and can successfully applied for contaminated wastewater treatment.

**Table 3:** Biodegradation of chlorophenols using different bioreactors

Bioreactor	Compound	Concentration (mg/L)	HRT (h)	Loading rate (mg/L/day)	Removal rate (%)	References
FBR	4-CP	99.13	24.4	97.5	98.7	Galíndez-Mayer et al. (2008)
PBR	4-CP	20	2.78	172	100	Kim et al. (2002)
CSTR	4-CP	20	4.17	115	100	Kim et al. (2002)
UASB	4-CP	40	12	80	88.3	Majumder and Gupta (2008)
ASU	4-CP	500	15	800	90	Kargi and Konya (2007)
ALR	2,4-DCP	7–103	6.25	26–394	100–88	Quan et al. (2003)
ALR	2,4-DCP	28.5	8	85.5	97.8	Quan et al. (2004)
PB-ALR	2,4,6-TCP	5.76	2.95	46.88	99.9	Gomez-De Jesus et al. (2009)
ALR	4-CP	400	24	400	99.8	This study

Activated sludge unit consisted of an aeration tank and a sludge settling tank (ASU); fluidized bed reactor (FBR); Packed bed reactor (PBR); continuous stirred tank reactor (CSTR); upflow anaerobic sludge blanket (UASB); airlift reactor (ALR); Packed bed airlift reactor (PB-ALR)

### Metabolites

During the biodegradation of 4-CP by airlift inner loop reactor, the spectroscopic and HPLC analysis indicates the presence of 5-CHMS in the effluent. At lower 4-CP concentration, no metabolites had been detected in the effluent. However, at higher 4-CP concentration, the presence of 5-CHMS (*λ*_max_ = 380 nm) was detected. This was also evidenced by the appearance of light yellow color in the reactor medium. 5-CHMS is the meta-cleavage product of the 4-chlorocatechol, the first intermediate of the 4-CP degradation pathway. When 4-CP concentration is above 300 mg/L, the calorimetric assay of the effluent sample shows the presence of 4-chlorocatechol. The presence of metabolites showed that the mixed consortium follows the meta-cleavage pathway for 4-CP biodegradation. There were no other metabolites detected in the effluent. These results were also confirmed in the batch study. The degradation of 4-CP via meta-cleavage pathway by the mixed consortium has been reported (Farrell and Quilty 1999; El-Sayed et al. 2009).

Single bacterial strain can degrade toxic compounds completely if provided the feasible environment and presence of primary growth substrate. However, sometimes pure strain does not possess or express all the enzymes required for complete mineralization of toxic compounds. They specifically express the enzymes that act on parent compounds and produce the intermediate metabolites. These intermediate metabolites remain in the medium unutilized, because the lack of enzymes requires in the metabolic pathway. Also, application of pure strains for in situ environment is impractical as the dominance of other strains over the special strains that are better fitted for the degradation of target compound. In nature or engineered system, microbial communities are responsible for the complete mineralization of the toxic compounds. The several species present act together in a coordinated way within the microbial community that leads to complete degradation of recalcitrant compounds. Also, mixed microbial consortium does not require the presence of other primary growth substrate such as phenol or carbon source for complete degradation or induction of enzymes for the transformation of target toxic compound. A better understanding of the role that microorganisms play in the biodegradation and removal of chlorophenol compounds from the environment could facilitate further research for effective implementation of bioremediation technologies for the contaminated sites. Specific treatment proficiency can enable removal of chlorophenols from the environment and subsequently their ecotoxicological effects.

## Conclusions

An airlift bioreactor was evaluated for the removal of 4-chlorophenol in synthetic wastewater using the mixed microbial consortium isolated from the dye industries. The results demonstrated that different parameters such as hydraulic retention time, initial substrate concentration, and peptone as biogenic substrate have profound effect on 4-CP removal efficiency. The bioreactor showed an excellent removal efficiency for 4-CP throughout the operation with greater than 98 % removal. The optimum hydraulic retention time for the bioreactor was found to be 24 h and below that the washout of the microbes observed. Effect of peptone showed that lower peptone concentration increases the removal efficiency; however, some peptone is necessary to maintain 4-CP removal efficiency. The maximum initial substrate concentration of 400 mg/L of 4-CP was efficiently removed up to 99 % at 24 h HRT and 0.2 g/L peptone. The presence of 5-CHMS at a higher 4-CP concentration in the bioreactor establishes that the mixed consortium follows the meta-cleavage pathway. The stability and efficiency showed by the mixed consortium for different operating conditions indicate its suitability for in situ bioremediation over a pure strain.

## References

[CR1] Alexander M (1999). Biodegradation and bioremediation.

[CR2] Arnow LE (1937). colorimetric determination of the components of 3,4-dihydroxyphenylalaninetyrosine mixtures. J Biol Chem.

[CR3] Arora P, Bae H (2014). Bacterial degradation of chlorophenols and their derivatives. Microb Cell Fact.

[CR4] ATSDR (2015) Comprehensive environmental response, compensation, and liability act (CERCLA), priority list of hazardous substances

[CR5] Atuanya EI, Chakrabarti T (2004). Kinetics of biotransformation of 2,4-dichlorophenol using UASB-reactor. Environ Monit Assess.

[CR6] Basak B, Bhunia B, Dutta S, Chakraborty S, Dey A (2013). Kinetics of phenol biodegradation at high concentration by a metabolically versatile isolated yeast *Candida tropicalis* PHB5. Environ Sci Pollut Res.

[CR7] Basak B, Bhunia B, Dey A (2014). Studies on the potential use of sugarcane bagasse as carrier matrix for immobilization of *Candida tropicalis* PHB5 for phenol biodegradation. Int Biodeterior Biodegrad.

[CR8] Chakraborty S, Basak B, Dutta S, Bhunia B, Dey A (2013). Decolorization and biodegradation of congo red dye by a novel white rot fungus *Alternaria alternata* CMERI F6. Biores Technol.

[CR9] Daniel V, Huber W, Bauer K, Suesal C, Mytilineos J, Melk A, Conradt C, Opelz G (2001). Association of elevated blood levels of pentachlorophenol (PCP) with cellular and humoral immunodeficiencies. Arch Environ Health.

[CR10] Durruty I, Okada E, González J, Murialdo S (2011). Multisubstrate monod kinetic model for simultaneous degradation of chlorophenol mixtures. Biotechnol Bioproc Eng.

[CR11] El-Sayed WS, Ismaeil M, El-Beih F (2009). Isolation of 4-chlorophenol-degrading bacteria, *Bacillus subtilis* OS1 and *Alcaligenes* sp. OS2 from petroleum oil-contaminated soil and characterization of its catabolic pathway. Aust J Basic Appl Sci.

[CR12] Farrell A, Quilty B (1999). Degradation of mono-chlorophenols by a mixed microbial community via a meta-cleavage pathway. Biodegradation.

[CR13] Field J, Sierra-Alvarez R (2008). Microbial degradation of chlorinated phenols. Rev Environ Sci Bio/Technol.

[CR14] Galíndez-Mayer J, Ramón-Gallegos J, Ruiz-Ordaz N, Juárez-Ramírez C, Salmerón-Alcocer A, Poggi-Varaldo HM (2008). Phenol and 4-chlorophenol biodegradation by yeast *Candida tropicalis* in a fluidized bed reactor. Biochem Eng J.

[CR15] Gomez-De Jesus A, Romano-Baez FJ, Leyva-Amezcua L, Juarez-Ramirez C, Ruiz-Ordaz N, Galindez-Mayer J (2009). Biodegradation of 2,4,6-trichlorophenol in a packed-bed biofilm reactor equipped with an internal net draft tube riser for aeration and liquid circulation. J Hazard Mater.

[CR16] Herrera Y, Okoh A, Alvarez L, Robledo N, Trejo-Hernández M (2008). Biodegradation of 2,4-dichlorophenol by a *Bacillus consortium*. World J Microbiol Biotechnol.

[CR17] Hollender J, Hopp J, Dott W (1997). Degradation of 4-chlorophenol via the meta cleavage pathway by *Comamonas testosteroni* JH5. Appl Environ Microbiol.

[CR18] Hu Z, Ferraina RA, Ericson JF, Smets BF (2005). Effect of long-term exposure, biogenic substrate presence, and electron acceptor conditions on the biodegradation of multiple substituted benzoates and phenolates. Water Res.

[CR19] IARC (1986). IARC monographs on the evaluation of the carcinogenic risk of chemicals to humans: some halogenated hydrocarbons and pesticide exposures.

[CR20] Kargi F, Konya I (2007). Para-chlorophenol containing synthetic wastewater treatment in an activated sludge unit: effects of hydraulic residence time. J Environ Manage.

[CR21] Karn SK, Chakrabarty SK, Reddy MS (2010). Pentachlorophenol degradation by *Pseudomonas stutzeri* CL7 in the secondary sludge of pulp and paper mill. J Environ Sci (China).

[CR22] Kim J-H, Oh K-K, Lee S-T, Kim S-W, Hong S-I (2002). Biodegradation of phenol and chlorophenols with defined mixed culture in shake-flasks and a packed bed reactor. Process Biochem.

[CR23] Majumder PS, Gupta SK (2008). Degradation of 4-chlorophenol in UASB reactor under methanogenic conditions. Bioresour Technol.

[CR24] Monsalvo VM, Tobajas M, Mohedano AF, Rodriguez JJ (2012). Intensification of sequencing batch reactors by cometabolism and bioaugmentation with Pseudomonas putida for the biodegradation of 4-chlorophenol. J Chem Technol Biotechnol.

[CR25] Murialdo SE, Fenoglio R, Haure PM, Gonzalez JF (2003). Degradation of phenol and chlorophenols by mixed and pure cultures. Water SA.

[CR26] Olaniran A, Igbinosa E (2011). Chlorophenols and other related derivatives of environmental concern: properties, distribution and microbial degradation processes. Chemosphere.

[CR27] Patel BP, Kumar A (2015). Optimization study for maximizing 2,4-dichlorophenol degradation by *Kocuria rhizophila* strain using response surface methodology and kinetic study. Desalin Water Treat.

[CR28] Patel BP, Kumar A (2016). Biodegradation of 2,4-dichlorophenol by Bacillus endophyticus strain: optimization of experimental parameters using response surface methodology and kinetic study. Desalin Water Treat.

[CR29] Poggi-Varaldo HM, Barcenas-Torres JD, Moreno-Medina CU, Garcia-Mena J, Garibay-Orijel C, Rios-Leal E, Rinderknecht-Seijas N (2012). Influence of discontinuing feeding degradable cosubstrate on the performance of a fluidized bed bioreactor treating a mixture of trichlorophenol and phenol. J Environ Manage.

[CR30] Quan X, Hanchang S, Yongming Z, Jianlong W, Yi Q (2003). Biodegradation of 2,4-dichlorophenol in an air-lift honeycomb-like ceramic reactor. Process Biochem.

[CR31] Quan X, Shi H, Zhang Y, Wang J, Qian Y (2004). Biodegradation of 2,4-dichlorophenol and phenol in an airlift inner-loop bioreactor immobilized with *Achromobacter* sp.. Sep Purif Technol.

[CR32] Sahinkaya E, Dilek FB (2006). Effect of biogenic substrate concentration on 4-chlorophenol degradation kinetics in sequencing batch reactors with instantaneous feed. J Hazard Mater.

[CR33] Sahinkaya E, Dilek FB (2007). Effect of feeding time on the performance of a sequencing batch reactor treating a mixture of 4-CP and 2,4-DCP. J Environ Manage.

[CR34] Shen DS, Liu XW, Feng HJ (2005). Effect of easily degradable substrate on anaerobic degradation of pentachlorophenol in an upflow anaerobic sludge blanket (UASB) reactor. J Hazard Mater.

[CR35] Visvanathan C, Thu LN, Jegatheesan V, Anotai J (2005). Biodegradation of pentachlorophenol in a membrane bioreactor. Desalination.

[CR36] Wang S-J, Loh K-C (1999). Facilitation of cometabolic degradation of 4-chlorophenol using glucose as an added growth substrate. Biodegradation.

[CR37] Wang Q, Li Y, Li J, Wang Y, Wang C, Wang P (2014). Experimental and kinetic study on the cometabolic biodegradation of phenol and 4-chlorophenol by psychrotrophic *Pseudomonas putida* LY1. Environ Sci Pollut Res.

[CR38] Ziagova M, Liakopoulou-Kyriakides M (2007). Kinetics of 2,4-dichlorophenol and 4-Cl-m-cresol degradation by *Pseudomonas* sp. cultures in the presence of glucose. Chemosphere.

[CR39] Zilouei H, Guieysse B, Mattiasson B (2006). Biological degradation of chlorophenols in packed-bed bioreactors using mixed bacterial consortia. Process Biochem.

